# Association of Cancer and the Risk of Developing Atrial Fibrillation: A Systematic Review and Meta-Analysis

**DOI:** 10.1155/2019/8985273

**Published:** 2019-04-14

**Authors:** Ming Yuan, Zhiwei Zhang, Gary Tse, Xiaojin Feng, Panagiotis Korantzopoulos, Konstantinos P. Letsas, Bryan P. Yan, William K. K. Wu, Huilai Zhang, Guangping Li, Tong Liu, Yunlong Xia

**Affiliations:** ^1^Tianjin Key Laboratory of Ionic-Molecular Function of Cardiovascular Disease, Department of Cardiology, Tianjin Institute of Cardiology, Second Hospital of Tianjin Medical University, Tianjin 300211, China; ^2^Department of Medicine and Therapeutics, Chinese University of Hong Kong, Hong Kong, SAR, China; ^3^Li Ka Shing Institute of Health Sciences, 30-32 Ngan Shing St, Chinese University of Hong Kong, Hong Kong, SAR, China; ^4^First Department of Cardiology, University Hospital of Ioannina, Ioannina, Greece; ^5^Second Department of Cardiology, Laboratory of Cardiac Electrophysiology, Evangelismos General Hospital of Athens, Athens, Greece; ^6^Department of Epidemiology and Preventive Medicine, Monash University, Clayton, Australia; ^7^Department of Anaesthesia and Intensive Care, Faculty of Medicine, The Chinese University of Hong Kong, Hong Kong, China; ^8^Department of Lymphoma, Tianjin Medical University Cancer Institute and Hospital, National Clinical Research Center of Cancer, Key Laboratory of Cancer Prevention and Therapy, Tianjin, China; ^9^Department of Cardiology, First Affiliated Hospital of Dalian Medical University, Dalian, China

## Abstract

**Aims:**

Previous studies have demonstrated epidemiological evidence for an association between cancer and the development of new-onset atrial fibrillation (AF). However, these results have been conflicting. This systematic review and meta-analysis was conducted to examine the relationship between cancer and the risk of developing atrial fibrillation.

**Methods:**

PubMed and Web of Science were searched for publications examining the association between cancer and atrial fibrillation risk published until June 2017. Adjusted odds ratios (ORs) or hazard ratios (HRs) and 95% CI were extracted and pooled.

**Results:**

A total of five studies involving 5,889,234 subjects were included in this meta-analysis. Solid cancer patients are at higher risk developing atrial fibrillation compared to noncancer patients (OR 1.47, 95% CI 1.31 to 1.66, *p* < 0.00001; *I*^2^ = 67%, *p*=0.02). The risk of atrial fibrillation was highest within 90 days of cancer diagnosis (OR 7.62, 95% CI 3.08 to 18.88, *p* < 0.00001) and this risk diminished with time.

**Conclusions:**

The risk of AF was highest within 90 days of cancer diagnosis. We should take into account the increased risk of atrial fibrillation development and, after this, study the embolic risk and potential indication of oral anticoagulation.

## 1. Introduction

Atrial fibrillation (AF) is the commonest cardiac arrhythmia observed in clinical practice and is associated with significant morbidity and mortality globally. Its prevalence is increasing in part due to an aging population [1], from ∼0.5% in those aged 40 years to 6–12% in those aged 85 years [2]. Apart from advancing age, independent predictors of AF include hypertension, obesity, diabetes, and smoking [1, 2]. Moreover, cancer remains the second most common cause of death in the United States. Several risk factors are known to contribute to both AF and cancer [3]. Thus, mechanistic studies have demonstrated a critical role of proinflammatory states in cancer [4]. A proinflammatory environment has also been linked to AF, as reflected by increases in serum inflammatory biomarkers such as C-reactive protein (CRP), interleukin- (IL-) 2, IL-6, and IL-8 [5]. However, evidence from epidemiological studies has been controversial [6–10]. Therefore, this systematic review and meta-analysis was conducted to examine the relationship between cancer and the risk of developing AF.

## 2. Methods

This meta-analysis of observational studies was in accordance with the Strengthening the Reporting of Observational Studies in Epidemiology (STROBE) statement [11].

### 2.1. Search Strategies

Two researchers (M. Y. and X. F.) systematically and independently searched the relevant studies from the following databases: PubMed (until June 2017) and Web of Science (from 1986 through June 2017). We used the following key words: “cancer,” “carcinoma,” “tumor,” and “atrial fibrillation.” The reference lists of the articles, conferences, and editorials were used to identify further relevant studies.

### 2.2. Inclusion Criteria

All observational studies reported on the epidemiological evidence for an association between solid cancer and AF were included in this meta-analysis. Because the aim of this meta-analysis is to investigate whether cancer patients are at an increased risk of developing AF in general population, study population that involved the patients who underwent surgery and chemotherapy were excluded. The inclusion criteria were articles (1) published in English language, (2) on human subjects, (3) that were case control, prospective or retrospective cohort study, (4) reporting the odds ratios (ORs) or hazard ratios (HRs) and the corresponding 95% confidence intervals (CIs) or data required for their calculations were provided, (5) assessing the association between AF and cancer, not survival of cancer or complication of AF. Where two articles with overlapping data were included, the articles with higher subjects were included.

### 2.3. Study Selection

Two researchers (M. Y. and X. F.) independently screened the titles and abstracts of the studies. Potential eligible studies were retrieved using the relevant inclusion criteria mentioned previously. Any disagreements or indeterminations between the two researchers were resolved through discussion or consultation with a third researcher (T. L.).

### 2.4. Data Extraction and Quality Assessment

Hazard ratio (HRs), odds ratio (ORs), and their 95% confidence intervals (CIs) for the association between cancer and AF were extracted from individual articles. If both unadjusted and adjusted ORs/HRs were reported, the adjusted ORs/HRs were preferentially used. And we priority to extracted multivariate adjusted ORs/HRs, not age- or gender-adjusted, to evaluate the risk of AF occurrence. The extracted data of each study included first author's last name, year of publication, geographic location of study, study design, total number of subjects, participants' age and sex, types of cancer, criterion for AF confirmation, follow-up duration, maximum adjusted covariates, HR or OR with 95% CI, and patients with heart failure, hypertension, and diabetic mellitus.

The Newcastle-Ottawa Scale (NOS) items, with total score of nine stars, were used to evaluate the quality of cohort or case control studies [12]. We defined the cohort or case control studies with NOS score ≥7 stars as high quality and NOS score <7 stars as low quality.

### 2.5. Statistical Analysis

The ORs with 95% CIs were used as the common risk estimates and then were pooled. The HR value using multivariate Cox proportional hazards model in the original research was directly considered as OR. Percent variability across studies attributable to heterogeneity beyond sampling error was evaluated using the *I*^2^ statistic, and *I*^2^ value of >50% represented moderate to high heterogeneity. The random effects model was used as it is better to explain heterogeneity between studies over the fixed-effects model. Subgroup analyses and sensitivity analysis were performed to identify the sources of heterogeneity. Subgroup analyses regarding study design (case control studies or cohort studies), study location (Europe and USA), and the methods for AF diagnosis (electrocardiogram and International Classification of Disease code) and time interval between cancer diagnosis and AF were performed. Sensitivity analysis was performed by omitting one study at a time to evaluate the influence of individual studies on the pooled results. The funnel plot was constructed to identify possible publication bias. *P* values of <0.05 (two-tailed) were considered statistically significant. Statistical analyses were performed using the Review Manager (RevMan) software (Nordic Cochrane Center; http://ims.cochrane.org/revman, version 5.3).

## 3. Results

**Figure 1:**
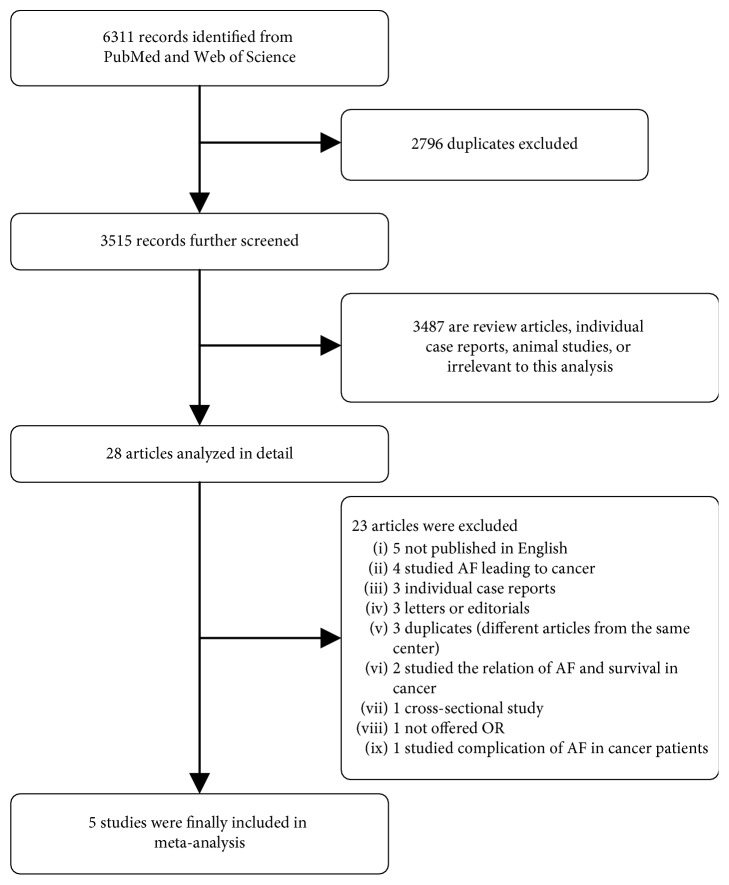
Flow diagram of the study selection process. AF = atrial fibrillation; OR = odds ratio.

A flow diagram of the data search and study selection is shown in Figure 1. Initially, 6311 records were identified from the PubMed and Web of Science databases. Of these, 2796 were duplicate studies and were excluded. The remaining articles were screened, and 3487 were subsequently excluded because they were review articles, animal studies, or irrelevant to this analysis. The 28 remaining studies were then reviewed in detail, and 23 of the 28 were excluded: study published in Italian, Russian, or Spanish language (*n*=5) [13–16]; study reported AF leading to cancer (*n*=4) [17–20]; individual case reports (*n*=3) [21–23]; letters [24, 25] or editorials [26] (*n*=3); different article from the same center [27, 28], and a more recent series was available [8], patients in this study [29] may be repeated in the study by Conen [10] (*n*=3); study evaluated the relation of AF and survival in cancer patients (*n*=2) [30, 31]; OR was not provided (*n*=1) [32]; cross-sectional study (*n*=1) [33]; and study investigated AF as a complication in cancer patients (*n*=1) [34]. Therefore, a total of five studies comprising 5,889,234 participants were included in our meta-analysis.

The characteristics of the included studies and their quality scores are shown in Table 1. Two were case control studies [7, 8], and three were cohort studies [6, 9, 10]. Two studies [6, 7] investigated only the association between AF and colorectal cancer, whereas three studies [8–10] examined colorectal cancer and also cancer of the breast, lung, and prostate. For the included cohort, the mean age ranged from 53 to 75 years and the proportion of male patients accounted for all patients ranged from 0% to 60%. Characteristics of the patients are presented in Table 2. NOS analysis showed that all included studies were of high quality.

**Figure 2:**
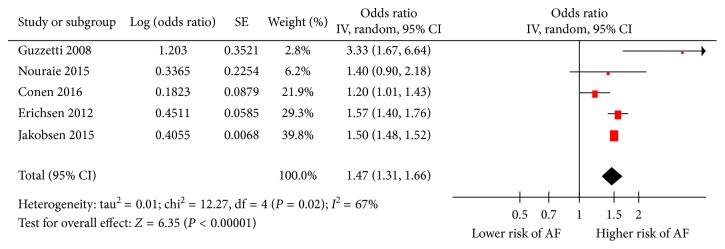
Forest plot for pooled odds ratio (OR) for the risk of atrial fibrillation (AF) in patients with any cancer. SE = standard error; IV = inverse variance.

Apart from one study [6] reporting no significant association between cancer and AF, the remaining four studies [7–10] consistently demonstrated a significant association between cancer and new AF risk. Overall, the summary estimate from the five separate estimates from the cohort or case-control studies united indicated that patients with cancer had an approximately 47% higher risk of AF compared to noncancer patients (OR 1.47, 95% CI 1.31 to 1.66, *p* < 0.00001; Figure 2). There was a significant heterogeneity across the studies (*I*^2^ = 67%, *p*=0.02).

**Figure 3:**
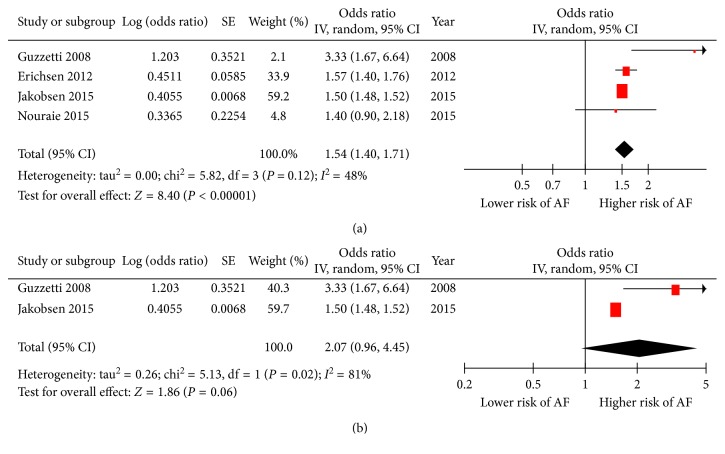
Outcomes for the risk of atrial fibrillation (AF) in patients with different cancer. (a) Forest plot of the odds ratio (OR) for pooled risk of atrial fibrillation (AF) in patients with colorectal cancer; (b) forest plot of the odds ratio (OR) for pooled risk of atrial fibrillation in patients with breast cancer. SE = standard error; IV = inverse variance.

Four of five studies reported OR of incidence of AF in colorectal cancer; the pooled effect sizes of these studies [6–9] (OR 1.54, 95% CI 1.40 to 1.71, *p* < 0.00001; heterogeneity: *I*^2^ = 48%, *p*=0.12; Figure 3(a)) showed that patients with colorectal cancer at a 54% higher risk of developing AF than those without colorectal cancer. Two of five studies offered OR of incidence of AF in breast cancer; in the pooled analysis of these studies [8, 9] (OR 2.07, 95% CI 0.96 to 4.45, *p*=0.06; heterogeneity: *I*^2^ = 81%, *p*=0.02; Figure 3(b)), we observed a prevalence of AF close to two times higher in breast cancer patients compared to those without breast cancer.

Subgroup analyses were subsequently performed to identify potential sources of heterogeneity. The details are shown in Table 3. Our pooled meta-analysis suggested that cancer is significantly associated with the occurrence of AF in both cohort [6, 9, 10] and case control [7, 8] studies with significant heterogeneity. As shown in Table 3, cancer was associated with an increased risk of AF in both studies originating from Europe [7–9] (OR 1.56, 95% CI 1.39 to 1.76, *p* < 0.0001, *I*^2^ = 65%) with significant heterogeneity as well as the United States [6, 10] (OR 1.22, 95% CI 1.04 to 1.44, *p*=0.01, *I*^2^ = 0%) without heterogeneity.

Subgroup analysis was also performed for the method of AF diagnosis. Meta-analysis of two studies using electrocardiography [8, 10] reported a higher risk of AF in cancer patients (OR 1.89, 95% CI 0.70 to 5.10, *p*=21, *I*^2^ = 87%) with significant heterogeneity. Meta-analysis of two studies that had used the International Classification of Diseases (ICD) [6, 7] also demonstrated elevated risk of AF (OR 1.56, 95% CI 1.39–1.74, *p* < 0.0001, *I*^2^ = 0%), and this was associated with minimal heterogeneity. Therefore, study location [6–10] and the method for diagnosing AF [6–8, 10] are both likely the origin of the heterogeneity in our main meta-analysis.

**Figure 4:**
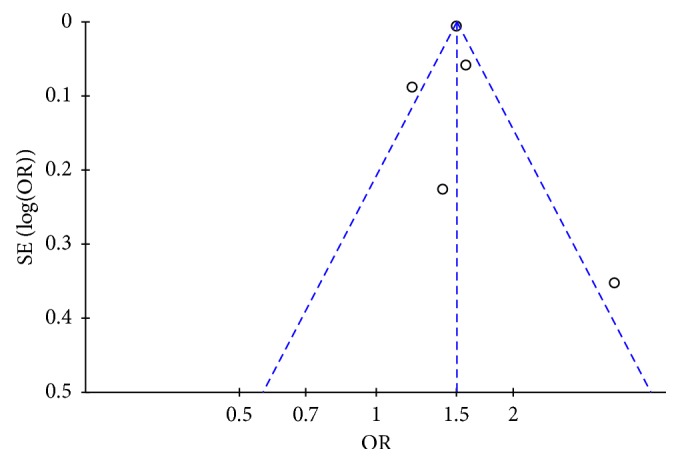
Funnel plot for the association between cancer and atrial fibrillation occurrence. SE = standard error; OR = odds ratio.

To examine temporal relationship between cancer and AF, ORs from two studies were further pooled [7, 10] for AF according to the time since cancer was first diagnosed, classified by time interval less than 90 days, 91 to 365 days, or more than 365 days. We found significantly increased risk of AF in cancer patients diagnosed less than 90 days (OR 7.62, 95% CI 3.08 to 18.88, *p* < 0.0001, *I*^2^ = 91%). Otherwise, pooled OR of incidence of AF was not significantly increased for longer time-points of 91 to 365 days (OR 1.06, 95% CI 0.90 to 1.25, *p*=0.46, *I*^2^ = 0%) or beyond 365 days (OR 0.97, 95% CI 0.71 to 1.34, *p*=0.87, *I*^2^ = 84%) (Table 3). Finally, sensitivity analysis by excluding one study at a time did not significantly alter the pooled OR. The results of the funnel plot for the association between cancer and AF was asymmetry, indicating that publication bias may be present, although the small number of studies made this somewhat difficult to interpret (Figure 4).

## 4. Discussion

Our systematic review and meta-analysis of five published observational studies suggests that subjects with newly diagnosed cancer had a significantly increased risk of AF during subsequent follow-up. There was significant heterogeneity observed between the included studies which were likely due to different methods of AF diagnosis. Interestingly, the increased risk of AF was only observed within the first 90 days after cancer diagnosis, and the risk was not significant after 1 year. Our study found substantial statistical heterogeneity in the pooled effect estimates. This is partly explicable by the use of different methods to detect AF in the individual studies. There are some data to support an increased risk of stroke after a cancer diagnosis, and one could hypothesize that this relationship between cancer and AF could account for part of the elevated risk of stroke if the AF went undetected.

Oncocardiology is a new field of clinical medicine that addresses the close link between cancer and cardiovascular diseases [35, 36]. Recent evidence showed that cancer is closely related to the development of AF. A number of pathophysiological mechanisms, such as inflammation and autonomic dysfunction, have been proposed to explain this link [5, 37]. Firstly, clinical studies have demonstrated elevations in proinflammatory markers in both AF and cancer. A case-control study showed that patients with atrial arrhythmia compared to those without atrial arrhythmia had higher levels of the inflammatory marker, C-reactive protein (CRP). Indeed, CRP levels were higher in persistent than paroxysmal AF patients [38]. Another large population-based cohort study reported that CRP was independently associated with the presence of AF and also predicted patients who were at increased risk of subsequently developing AF [39]. Marcus et al. reported that CRP levels in patients with atrial flutter that were initially elevated fell after successful ablation [40]. These findings are consistent with notion that inflammation plays a key role in structural and electrophysiological modeling that underlies arrhythmic substrates [41]. Similarly, cancer is associated with a proinflammatory state [42]. Thus, CRP levels were significantly elevated in colorectal [43] and breast [44] cancer patients compared to controls. However, in the included studies, data on cancer staging were not available, which could alter the degree of inflammation and the risk of AF.

The second factor is dysfunction of the autonomic nervous system. Altered balance of sympathetic versus parasympathetic activity has been associated with AF [45], and patients with cancer also show some evidence of altered autonomic activity or dysfunction [46]. Pain and emotional or physical stress in cancer may increase sympathetic nervous activity and predispose to atrial fibrillation [47]. Therefore, the patients who diagnosed with cancer underwent mental stress and sympathetic challenge during the first 90 days of receiving such anxiety provoking news which may have predisposed the patients to triggers of AF. Besides, Faber et al. reported that subclinical thyroid diseases seemed to change the structure and function of heart with subsequent alters in morbidity and mortality [48]. And the outcome of another analysis demonstrates that patients with clinical or subclinical hyperthyroidism are at increased risk of AF [49]. A hypothesis concerns that tumors may release thyroid hormones such as thyroid-stimulating hormone (TSH) and triiodothyronine (T3) [8]. Thus, it is possible that cancer enhances the incidence of AF through the abnormal production of thyroid hormones-like peptides [50]. These findings suggest altered autonomic activity in occult cancer, suggesting that undiagnosed cancer may precede AF. Finally, the epidemiological link between AF and cancer may be due to shared risk factors, diagnostic bias, undiagnosed occult cancer leading to AF, and anticoagulation unmasking cancer.

There are several potential limitations of our meta-analysis. Firstly, the data on the epidemiological evidence for the relationship between new-onset cancer and the risk of AF are sparse, and only a few studies have addressed this issue. And given that the total number of studies was small, we included a letter [28] and an abstract of conference [9] to maximize the use of available data. Secondly, some studies only presented the sex- or age-adjusted ORs/HRs, so not all ORs/HRs used in this analysis were extracted from multivariate analysis. This may have introduced a degree of random error in our pooled analysis. Finally, the epidemiological evidence for an association between cancer and risk of AF is mainly investigated in this study, and we will perform further study to uncover the potential relationship between surgery or chemotherapy and development of atrial fibrillation.

In summary, this meta-analysis demonstrates that cancer is associated with atrial fibrillation that was significant within the first three-month period. These findings would prompt us to suggest that AF patients should be screened for occult cancer. Future studies are needed to examine the potential mechanisms linking cancer to AF, and further analyses that can examine the association between AF and subsequent cancer-related mortality may exclude to possibility of diagnostic bias.

## Figures and Tables

**Table 1:** Characteristics of the five studies included in this meta-analysis.

First author and year	Location	Period of enrollment	Study design	Total patients, *N*	Cancer type	Incident cases of AF, *N* (%)	AF type	Criterion for AF diagnosis	Covariates in adjusted model	Follow-up (year)	Quality score
Guzzetti 2008 [8]	Italy	1987–2004	Case-control	1868	Colorectal and breast cancer	49 (2.6)	New-onset AF	Routine presurgery ECG	Age, sex	NA	8
Erichsen 2012 [7]	Denmark	1999–2006	Case-control	311593	Colorectal cancer	NA	AF/Flutter	According to the ICD	Age, sex, country	NA	8
Jakobsen 2015 [9]	Denmark	2000–2012	Prospective cohort	5539824	All types of cancer	NA	New-onset AF	NA	Age, sex	12	7
Nouraie 2015 [6]	USA	2000–2012	Retrospective cohort	1258	Colorectal cancer	93 (7.4)	New-onset AF	According to the ICD	Age, HTN, HF, DM, alcohol, tobacco	NA	8
Conen 2016 [10]	USA	1993–2013	Prospective cohort	34691	All types of cancer	824 (2.3)	New-onset AF	Electrocardiographic AF documentation or a medical report that documented a diagnosis of AF	Age, EDU, race, height, BMI, HTN, DM, smoking, HC, alcohol, physical activity, CHF, MI, stroke	19.1	9

AF = atrial fibrillation; BMI = body mass index; CHF = congestive heart failure; CRP = C-reactive protein; DM = diabetes mellitus; ECG = electrocardiography; EDU = educational lever; HC = hypercholesterolemia; HDL-c = high-density lipoprotein cholesterol; HF = heart failure; HTN = hypertension; ICD = International Classification of Diseases; LVH = left ventricular hypertrophy; MI = myocardial infarction; NA = not applicable; SBP = systolic blood pressure; TC = total cholesterol.

**Table 2:** Patient characteristics of the five studies.

First author and year	AF in CA/NO-CA (%)	Mean age, CA/NO-CA (year)	Male sex, CA/NO-CA (%)	Diabetes, CA/NO-CA (%)	Hypertension, CA/NO-CA (%)	Smoking, CA/NO-CA (%)	Cardiovascular disease, CA/NO-CA (%)	BMI, CA/NO-CA	Race, CA/NO-CA (%)	Education, high school, CA/NO-CA (%)
Guzzetti 2008 [8]	3.6/1.6	63.1 ± 12.6/63.6 ± 9.3	25.7/53.0	NA	NA	NA	NA	NA	NA	NA
Erichsen 2012 [7]	NA	75 (70–79)/74 (70–79)	54.1/54.1	7.1/4.5	3.3/2.3	NA	37.7/21.7	NA	NA	NA
Jakobsen 2015 [9]	NA	66.5 (66.5–66.5)/NA	47.4/NA	NA	NA	NA	NA	NA	NA	NA
Nouraie 2015 [6]	10.1/5.4	67 (58–78)/56 (48–69)	47/51	33/29	59/50	27/31	14/13	NA	NA	NA
Conen 2016 [10]	3.8/2.3	55 (50–61)/53 (49–58)	0/0	3.2/2.6	30.0/25.8	53.3/47.5	NA	25.1 (22.6–28.9)/24.9 (22.5–28.3)	White: 96.5/94.9	43.4/44.4

CA = cancer group; NA = not applicable; NO-CA = noncancer group.

**Table 3:** Subgroup analysis of the association between cancer and atrial fibrillation.

	Subgroup	Number of studies	Meta-analysis			Heterogeneity	
			OR	95% CI	*p* value	*I* ^2^ (%)	*p* value
Study design	Case control	2	2.11	1.03–4.34	0.04	77	0.04
	Cohort	3	1.38	1.15–1.64	0.0004	69	0.04

Study location	Europe	3	1.56	1.39–1.76	<0.0001	65	0.06
	USA	2	1.22	1.04–1.44	0.01	0	0.52

Criterion for AF diagnosis	ECG	2	1.89	0.70–5.10	0.21	87	0.005
	ICD	2	1.56	1.39–1.74	<0.0001	0	0.62

Time interval between cancer diagnosis and AF	≤90 days	2	7.62	3.08–18.88	<0.0001	91	0.0009
	91–365 days	2	1.06	0.90–1.25	0.46	0	0.48
	>365 days	2	0.97	0.71–1.34	0.87	84	0.01

AF = atrial fibrillation; CI = confidence interval; ECG = electrocardiogram; ICD = International Classification of Disease; OR = odds ratio.
